# Exploring the Association Between Sialic Acid and SARS-CoV-2 Spike Protein Through a Molecular Dynamics-Based Approach

**DOI:** 10.3389/fmedt.2020.614652

**Published:** 2021-01-13

**Authors:** Leonardo Bò, Mattia Miotto, Lorenzo Di Rienzo, Edoardo Milanetti, Giancarlo Ruocco

**Affiliations:** ^1^Center for Life Nanoscience, Istituto Italiano di Tecnologia, Rome, Italy; ^2^Department of Physics, Sapienza University, Rome, Italy

**Keywords:** sialic acid, SARS- CoV-2, molecular dynamics, molecular binding, entry mechanism

## Abstract

Recent experimental evidence demonstrated the capability of SARS-CoV-2 Spike protein to bind sialic acid molecules, which was a trait not present in SARS-CoV and could shed light on the molecular mechanism used by the virus for the cell invasion. This peculiar feature has been successfully predicted by *in-silico* studies comparing the sequence and structural characteristics that SARS-CoV-2 shares with other sialic acid-binding viruses, like MERS-CoV. Even if the region of the binding has been identified in the N-terminal domain of Spike protein, so far no comprehensive analyses have been carried out on the spike-sialic acid conformations once in the complex. Here, we addressed this aspect performing an extensive molecular dynamics simulation of a system composed of the N-terminal domain of the spike protein and a sialic acid molecule. We observed several short-lived binding events, reconnecting to the avidic nature of the binding, interestingly occurring in the surface Spike region where several insertions are present with respect to the SARS-CoV sequence. Characterizing the bound configurations via a clustering analysis on the Principal Component of the motion, we identified different possible binding conformations and discussed their dynamic and structural properties. In particular, we analyze the correlated motion between the binding residues and the binding effect on the stability of atomic fluctuation, thus proposing regions with high binding propensity with sialic acid.

## 1. Introduction

The epidemic of SARS-CoV-2, a novel strain of β-coronavirus first documented in late 2019 (1, 2), has rapidly become pandemic. Before the appearance of SARS-CoV-2, four other β-coronaviruses were reported to infect humans. In particular, two of them have already caused severe epidemics worldwide: SARS-CoV, which cause the Severe Acute Respiratory Syndrome (SARS) in 2003 (3, 4), and MERS-CoV, the Middle East Respiratory Syndrome (MERS), first isolated in 2012 (5). The other human β-coronaviruses, *OC*43 and *HKU*1, are known to circulate in the human population for many years and cause a mild seasonal illness (6). On the contrary, SARS-CoV-2 mainly affects the lower respiratory system, causing pneumonia, similarly to SARS-CoV and MERS-CoV. Besides, the virus has been found also in other regions of the body, like the gastrointestinal system or the central nervous system where its effects are still under scrutiny (2, 7–9). Moreover, currently no approved vaccines are present although different antiviral compounds have been proposed (10–13).

From a molecular point of view, the Spike protein of CoVs, protruding from the viral membrane, not only plays a crucial role as a fundamental structural protein, but it also is essential for the interaction between CoV systems and host cells (14). Structurally, the spike protein is found in the trimeric complex, each chain composed of two sub-units: S1 and S2. In SARS-CoV, MERS-CoV, and SARS-CoV-2, the Receptor Binding Domain (RBD), located in the S1 domain, is responsible for viruses interaction with receptors on the host cell surface (15). On the other hand, the S2 subunit is responsible for the fusion between the virus and host membrane, causing the viral genome to penetrate the host cells cytoplasm (16).

To explain the entry molecular mechanism of SARS-Cov-2, a great effort is being spent in the study of the interactions at the molecular level between the virus and the host cells. In recent months it has been shown, through *in vivo* experiments, that SARS-CoV-2 uses the human Angiotensin-Converting Enzyme 2 (ACE2) receptor as primary entry channel (17), similarly to what done by SARS-CoV (18, 19).

Interestingly, the interaction with ACE2 involves the C-terminal domain of SARS-CoV-2 Spike protein, whose amino acid sequence is well conserved with respect to SARS-CoV homologous one (20). Conversely, the N-terminal domain presents some insertions, and these additional surface regions could be used by the virus to bind other cell receptors, so constituting an additional cell entry mechanism (17).

It has been actually demonstrated that several coronaviruses employ sialoglycan-based receptors as key component (21). These viruses bind to cell surface components containing N-acetyl-9-O-acetylneuraminic acid and this interaction is essential for the initiation of an infection (22). In particular, the structural details of the interaction between MERS-CoV Spike protein and a sialic acid molecule has been recently published (23). Comparing the structure of SARS-CoV-2 and MERS Spike proteins, we recently demonstrated that the insertions in the N-terminal domain confer to the solvent-exposed SARS-CoV-2 Spike a structure very similar to the region of the MERS-CoV Spike protein known to interact with sialic acid molecules (24).

These findings suggest that also SARS-CoV-2 gained the capability to bind host glycans, and recent computational and experimental literature confirms this hypothesis.

Indeed, while clinical investigations highlight that the replication of the virus does not take place only in the lower respiratory tract (25), Baker et al. (26) experimentally observed the binding between the NTD of the SARS-CoV-2 spike protein and sialic acid molecules using a glyco-nanoparticle platform.

Although no experimental data are available on the location of the binding region, computational works provided predictions of the possible involved residues. In particular, Robson et al. (27) used a new predictive algorithm to assign to each residue a sialic acid binding propensity. Notably, the regions around residues 58–70 and 255–265 on the N-terminal domain of the Spike display high scores toward sialic acid binding. Awasthi et al. (28) instead used docking techniques and simulations to evaluate the binding affinities of SARS-CoV-2 with different sialic acid molecules. They found that the flexibility of the Leu244-Gly261 region seems to confer the Spike the ability to bind a wide variety of sialosides.

Here, we present a computational structural biology study based on extensive molecular dynamics simulation aimed to further investigate the binding between SARS-CoV-2 Spike N-terminal domain (NTD) and a sialic acid molecule, in order to blindly highlight the spike region most prone to interact with glycans. We firstly perform a molecular dynamics simulation of the trimeric form of the spike protein, underlining how the inter-chain interactions influence very slightly the overall motion of each N-terminal domain. Thus, we run a molecular dynamics simulation with a sialic acid molecule, to blindly investigate the protein surface pocket preferentially contacted by the glycan. Our analyses assign to each spike residue a binding propensity score, based on the time that residue spends in interaction with sialic acid, identifying the sialic acid most preferred residues. Moreover, we executed a Principal Component Analysis on the coordinates of the interacting atoms belonging both to Spike protein and sialic acid molecule when these two molecules are bound, aiming to identify the principal conformations explored by the molecular complexes. We thus identified 5 binding modes occurring between these two molecules, involving five possible regions on the NTD, and we investigated if the presence of the glycan stabilize such regions.

These results shed light on this important molecular interaction, that could provide an additional cell invasion mechanism to the virus possibly explaining the impressive rate of infection showed by SARS-CoV-2.

## 2. Results and Discussion

To investigate protein-ligand interactions at the atomic level, molecular dynamics simulation (MD) with explicit solvent is a powerful computational approach because it allows us to take into account the exploration of the conformational space, as well as the solvent and entropic effects in a physically consistent manner (29), which are weakly considered in standard docking approaches. A crucial limitation of standard MD approaches is the exhaustiveness of the phase space sampling: in particular, when the energy barrier between the two states is too high, the study of the transition process is infeasible because the timescales of such transitions exceed those accessible to MD (30).

However, virus-glycan associations are typically characterized by low affinity and so they are inherently dynamic (31). Moreover, they are characterized by a high diffusion capacity of the molecule in water and by a few degrees of freedom. For these reasons, we studied the binding between spike protein of the SARS-CoV-2 and the sialic acid molecule through an extensive and completely unbiased MD simulation, intending to observe a spontaneous binding between the ligand and the protein.

**Figure 1:**
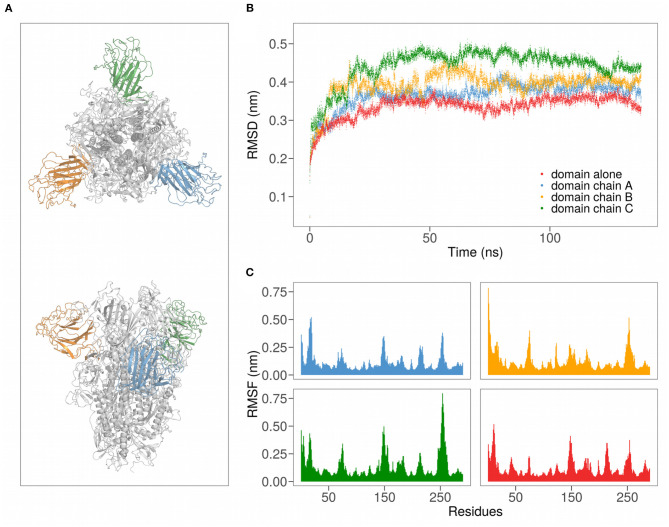
**(A)** Cartoon representation of the SARS-CoV-2 spike protein in trimeric form. Different colors highlight the three domains. **(B)** Root mean squared deviation (RMSD) as a function of time for the N-terminal regions of SARS-CoV-2 in the trimeric form (chains A, B, and C) and of chain A alone. **(C)** Root-mean squared fluctuation (RMSF) for all the 290 residues of the analyzed domains.

To keep the computational costs low, increasing at the same time the simulation time to have a greater probability of observing the interaction between protein and ligand, we used only the N-terminal domain (NTD) of the spike protein. Indeed, since both theoretical prediction and early experimental validations indicate that the sialic acid-binding region is localized in the NTD of the spike protein, we firstly compare the free motion of the domain (residues 1–290) when inserted in the trimeric spike complex and alone in water using 140 ns long MD trajectories (see section 4 for details and Figure 1A for a graphical representation).

### 2.1. Analysis of the Motion of SARS-CoV-2 N-Terminal Domain

To describe the motion of the N-terminal Spike domain, we performed two MD simulations, one with the domain alone in the water and one with the trimeric form of Spike.

We thus evaluated both the root mean square deviation (RMSD) and the root mean square fluctuation (RMSF) of the backbone carbons of the Spike N-terminal domain (see Figures 1B,C). Interestingly, in both the simulations the regions of the domain that present higher mobility (high RMSF) are formed by residues forming structural loops (see Table 1). Notably, these high motile regions are located on the Spike SARS-CoV-2 insertions with respect to SARS-CoV homolog.

**Table 1:** SARS-CoV-2 area under the curve values for the identified regions of high values of contact probability (peaks in Figure 2C).

**Residues range**	**Area**	**Peak**
1–23	0.117	Peak-1S
56–78	0.132	Peak-2S
140–165	0.153	Peak-3S
178–191	0.153	Peak-4S
241–265	0.194	Peak-5S

Remarkably, both descriptors behave similarly when the domain is alone in water (red in the Figure) or is inserted in the trimeric complex (different chain domains are represented in blue, orange, and green, respectively). In particular, the RMSD curves show the same trend, with about the same time to reach equilibrium, when both the single domain and the trimeric complex are considered. More specifically, the average RMSD value at equilibrium (after 30 ns of the simulation) of the N-terminal domain alone in solution is very similar to that of the same domain belonging to the chain A of the trimer, meaning that the interdomain interactions are not essential for the overall fold maintenance.

On the other hand, the comparison between the residues RMSF obtained when the domain is simulated alone or in trimer shows that the most fluctuating and least fluctuating regions are conserved. Indeed, the average Pearson correlation coefficient between the RMSF values of the three domains of the trimer and the RMSF values of the domain alone is 0.63. Looking at the correlation of the motions between residues, we underlined how even the residues combined motions are conserved when the domain is simulated alone (see Supplementary Material).

Taken together, these results indicate that the inter-chain interactions do not notably modify the motion of the Spike N-terminal domain. This allows us to simulate only the N-terminal domain, thus significantly reducing the computational cost of our simulations.

### 2.2. Molecular Simulation of the Binding Between SARS-CoV-2 and a Sialic Acid Molecule

We then performed a long molecular dynamics simulation of the N-terminal domain of SARS-CoV-2 in the presence of a sialic acid molecule (see section 4 for details), in order to observe a blind binding. To keep the computational cost of the simulation low and to avoid ligand-ligand interactions, we setup the system with a Spike N-terminal domain and a single sialic acid molecule.

**Figure 2:**
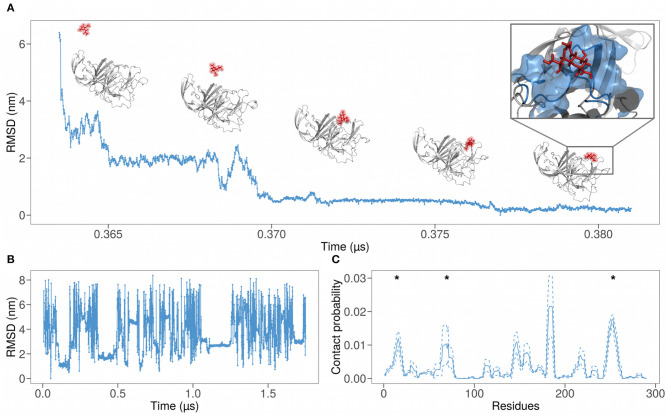
**(A)** Root mean square deviation (RMSD) as a function of time of the atomic positions of SARS-CoV-2 spike N-terminal domain and a sialic acid molecule using the final bound configuration as reference. The progressive nearing of the sialic acid molecule to the binding region can be seen also via the snapshots above the graph. **(B)** RMSD of the sialic acid molecule atomic positions as a function of simulation time. We used the first bound configuration as a reference. **(C)** Probability for each residue of the SARS-CoV-2 N-terminal domain to interact with the sialic acid molecule, obtained from a 1.75 μs long molecular dynamic simulation. The solid line represents the contact probability averaged over the six near-neighbor of each residue, while dashed lines show the mean value plus or less one standard deviation. Asterisks represent the regions of the binding predicted in (24).

During the 1.75μs of the evolution, we observed various binding (and unbinding) events. In particular, each event consists of a progressive nearing of the sialic acid to the protein until a binding takes place. Figure 2A displays some snapshots of one of the observed binding events, where the positions of the sialic acid molecule are measured in terms of RMSD, using the final bound configuration as a reference. As intuitively, we observe a progressive decrease of the RMSD, until the sialic acid binds to the protein.

As testified by the various constant stretch in Figure 2B, the binding is not unique, probably due to the aviditic nature of the interaction (31). Therefore, we defined a measure of local binding propensity, based on the number of contacts each residue does with sialic acid during the time evolution of the simulation. Indeed, for each residue, we calculate the fraction of frames in which the sialic acid center of mass is located at a distance lower than 6 Å to any heavy atoms of the residue.

In Figure 2C, the dynamics binding propensities for each residue are shown. We performed a smoothing procedure for each residue, averaging the contact probability of the examined residue (solid lines) on a sequence window of 7 residues centered on it. Dashed lines represent the fluctuations around the mean.

The contact probability as a function of the residues shows well-defined peaks. This means that the binding with sialic acid involves specific regions. The sum of all the areas under the most pronounced peaks, whose residue ranges are reported in Table 1 and represent the regions with the highest contact frequency, consists of the 75% of the total contacts occurring between spike and sialic acid.

Asterisks in Figure 2C mark the regions of SARS-CoV-2 that show a high shape similarity with the MERS-CoV binding region, as explained in (24). Notably, these three out of five peaks coincide with the previously identified region, while the peak in the range 140–165 is in close proximity to the predicted region.

### 2.3. Possible Binding Conformations

To characterize the different observed binding events, we investigated the structural conformations of the formed complexes. To do so, we performed a principal component analysis (PCA) on the spatial coordinate of the heavy atoms of both the 111 interacting residues of the spike domain and the sialic acid for uniform sampling of the molecular dynamics trajectory. Since we found not an unique binding region, we looked for a compact way to study and isolate the different binding regions on the spike protein. The PCA allows us to compactly represent each molecular dynamics frame as a point in essential plane, where the difference with the other possible bound conformation is maximum. In this way we are sure that the clustering procedure groups conformations that are similar focusing only on the position of the interacting atoms, considering both the protein and the ligand.

**Figure 3:**
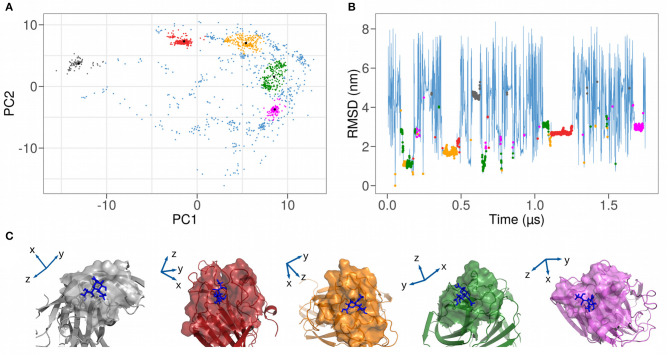
**(A)** Representation as a function of the 2 Principal Components of the coordinates of the heavy atoms of both the 111 spike interacting residues and the sialic acid. A clustering analysis (described in section 4) isolates five distinct clusters, highlighted with different colors in the plot. **(B)** Time evolution of the root mean square deviation (RMSD) of the atomic positions of SARS-CoV-2 spike N-terminal domain and a sialic acid molecule using the first bound configuration as a reference. Bound frames are colored according to the 5 clusters found in **(A)**. **(C)** Cartoon representations of the five different complexes between SARS-CoV-2 spike N-terminal domain and sialic acid molecule. Solvent-exposed molecular surfaces in the binding region are highlighted.

Figure 3A shows the projection of the sampled bound configurations in the plane identified by the first two principal components, which represent 69% of the total variance. Since each binding pose is characterized by a different set of atomic coordinates, this protocol allows us to group similar structures in the essential space of the first two principal components. The different binding configurations form clusters in the plane, which can be isolated upon performing a clustering analysis with a density filter (see details in section 4). We end up with five clusters, highlighted in different colors in Figure 3A.

In Table 2, we report, for each identified cluster, the percentage of its population with regard to the total bound frames of the trajectory. For example, the most frequent binding region is cluster 2, with a percentage of all other binding conformations of 32%.

**Table 2:** Summary of the relevant features for the five clusters identified in Figure 3A: percentage of bound conformations assigned to each cluster, list of residues in interaction with the sialic acid molecule, mean RMSF in free and bound state of the residues associated to each cluster.

**Cluster population (%)**		**Residues**	** ${\bar{\text{RMSF}}}_{\text{free}}$ **	** ${\bar{\text{RMSF}}}_{\text{bound}}$ **
Cluster 1	8.91	Phe 32, Thr 33, Phe 59, Pro 217, Pro 218, Gln 219, Phe 220	0.11 ± 0.01	0.10 ± 0.01
Cluster 2	31.93	His 66, Ala 67, Ile 68, Val 70, Ser 71, Gly 72, Gln 183, Gly 184, Asn 185, Phe 186, Val 213, Arg 214, Gly 261	0.25 ± 0.03	0.17 ± 0.02
Cluster 3	22.52	Lys 147, Gly 181, Gln 183, Tyr 248, Leu 249, Thr 250, Pro 251, Gly 252, Asp 253	0.44 ± 0.07	0.37 ± 0.06
Cluster 4	22.42	Tyr 144, Tyr 145, His 146, Trp 152, Met 153, Tyr 248, Pro 251, Gly 252, Asp 253, Ser 254	0.38 ± 0.08	0.47 ± 0.11
Cluster 5	14.21	Cys 15, Val 16, Phe 140, Glu 156, Arg 158, Arg 246	0.13 ± 0.02	0.09 ± 0.01

The groups obtained via the PCA analysis can be investigated following the time evolution of the simulation. Indeed, we show in Figure 3B the RMSD plot where each bound state was colored according to the coloring scheme defined with the PCA clustering and in Figure 3C an illustration of the 5 found binding modes. This RMSD representation allows the investigation of the duration time of each binding event. Indeed, even if the population of 2 clusters can be comparable, it is possible to have many unstable or few stable events. For example, cluster 2 seems to be more stable than cluster 4, since it is characterized by a unique and long binding event, even if the simulation time should be considerably increased to have a reliable conclusion.

### 2.4. Protein-Ligand Binding Characterization

The principal component analysis allows us to identify binding conformations between the N-terminal domain of the SARS-CoV-2 spike protein and the sialic acid molecule. Furthermore, selecting the most representative structure of each cluster and analyzing the protein-ligand inter-molecular contacts, we define 5 possible binding sites. Identified the interacting residues involved in the binding sites, we investigated their dynamic and structural properties, analyzing, on one hand, the correlation of motion between the residues during the unbound simulation time and on the other hand the effect of the binding in terms of structural stabilization.

**Figure 4:**
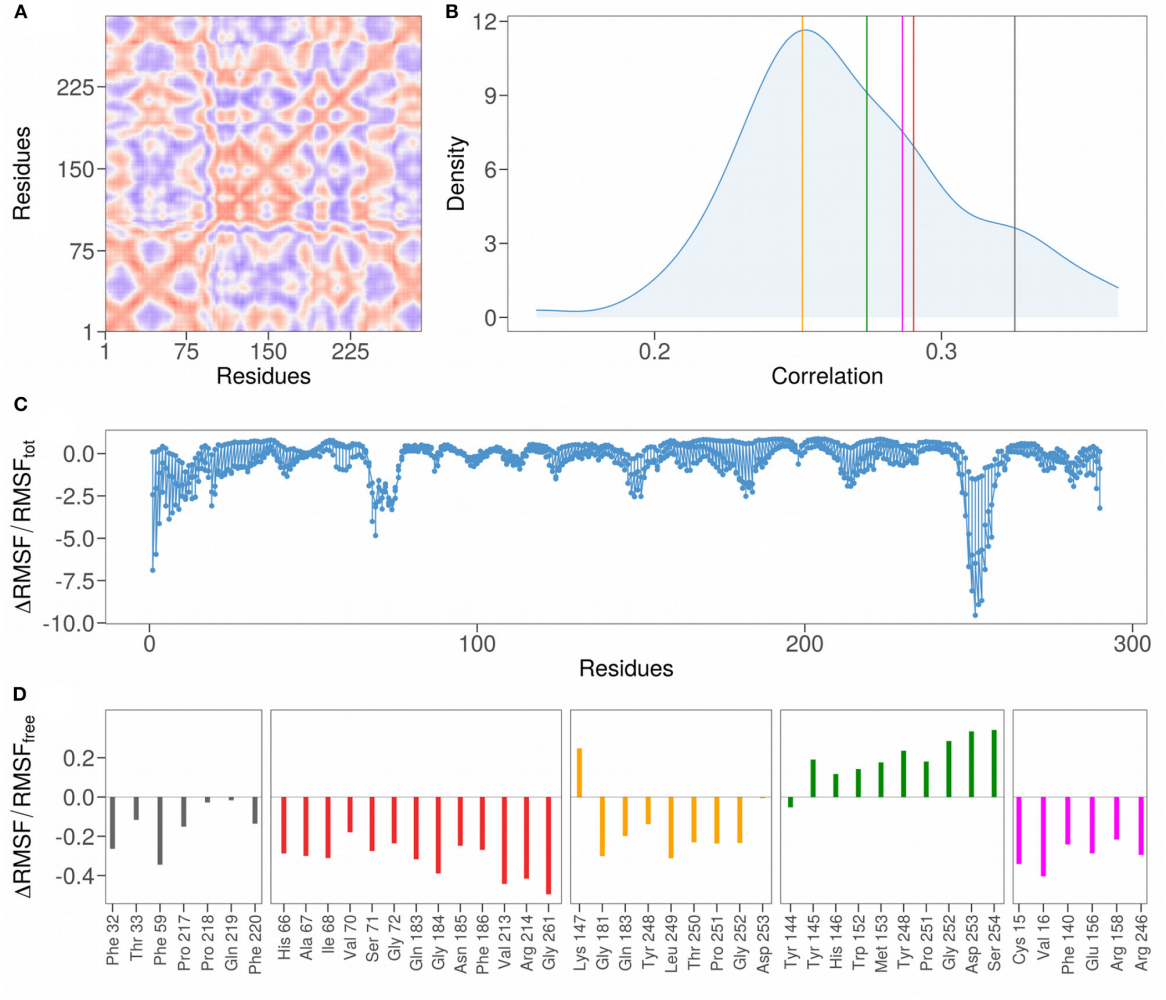
**(A)** Pearson pairwise correlation matrix of the motion of the 290 residues constituting the N-terminal domain of the SARS-CoV-2 spike protein. The correlation between a couple of residues is obtained averaging the correlation on the motion in every direction. Colors range from blue to red as the correlation passes from −1 to 1. **(B)** Density distributions of the mean correlation coefficients obtained grouping residues inside a sphere of radius 8 Å centered on each residue of the 111 residues forming the NTD loop regions. Vertical colored lines mark the mean values of the correlation of the couples of residues found in interaction with the sialic acid residue in the five clusters identified in Figure 3. **(C)** Difference in the root mean square fluctuations (RMSF) for each of the 290 residues comprising the N-terminal domain, computed using the whole dynamics (RMSF_tot_) or only the configurations where the sialic acid molecule is not bound to the protein (RMSF_free_) divided by RMSF_tot_. **(D)** Difference in RMSF for each of residues comprising the binding sites found in Figure 3, computed using the bound configurations (RMSF_bound_) or the free ones RMSF_free_ and divided by RMSF_free_.

The correlation analysis between the residues of the N-terminal domain, shown in Figure 4A, allows the identification of the sub-regions characterized by a high correlated motion in the unbound state, meaning that there is a dependence between the motion of these residues implying a possible functional role. More interestingly, in order to investigate the role of residues identified in interaction, we compare their distribution of Pearson correlations with those regarding set of patches centered on the residues of the loops (shown in Table 1). In particular, in order to compare the average correlation between the residues of each proposed binding site with a set of random non-interacting surface regions, we define 111 random patches, centered each time on one of the loops residues and composed by the residues closer than 8 Å to it. The mean correlation of the residues belonging to the randomly selected patches is 0.27 (Figure 4B, blue distributions), while for each binding site identified by the PCA the correlations are 0.33, 0.29, 0.26, 0.27, and 0.29 for the cluster 1, cluster 2, cluster 3, cluster 4, and cluster 5, respectively (Figure 4B, colored lines). Interestingly, the correlations of the motion of the residues of both cluster 3 and cluster 4 (which are indicated in orange and green in the figures) are less than or equal to the average correlation of residues belonging to non-interacting patches. On the other hand, clusters 1, 2, and 5 are characterized by correlations higher than the mean of the correlations of the random patches.

We also analyzed the variation of the mobility of the interacting residues before and after the binding with the ligand. For this purpose, we calculated the percentage difference of the atomic fluctuation between the bound and unbound state for each N-terminal domain residue (Figure 4C). Notably, the region with the highest values, containing the Tryptophan 258, is characterized by high structural stabilization upon binding with the ligand, and the same region was predicted as a possible interacting region by (24) and (27). Similarly, two other regions show a high level of stabilization after binding: the N-terminal loop region and the region centered on Lysine 77. Note that the N-terminal loop is characterized by high mobility (high RMSF values) in the simulation where the NTD is considered free in solution, as can be seen in Figure 1C. This analysis shows that the N-terminal loop could play a role in binding given its evident stabilization upon binding.

More specifically, we here also analyze the stabilizing effect of binding with the ligand by considering only the residues of the 5 binding sites identified. Similarly to what was found with the correlation analysis, clusters 1, 2, and 5 also show a highly stabilizing effect upon binding with the sialic acid molecule (Figure 4D). Interestingly, the residues belonging to cluster 4 (indicated in green) are characterized by a higher fluctuation of atomic mobility in their bound state with the ligand. The binding region with the highest degree of stabilization is that of cluster 2 (in red), having the average percentage difference in RMSF before and after binding of −0.32, in comparison with −0.15, −0.16, 0.2, and −0.30 for cluster 1, cluster 3, cluster 4, and cluster 5, respectively.

Thus, the analysis of the RMSD (Figure 2B) shows that the kinetics of the binding between a ligand and the binding site of cluster 4 is much faster than that between sialic acid and cluster 2 (the duration of the cluster 4 binding events is lower than that with cluster 2). This behavior could be explained by the after binding high atomic fluctuations of the cluster 4 residues and suggests the exclusion of this region as a possible region of interaction with sialic acid. More detailed data about the life-time of binding events can be found in Supporting Information. A more accurate study would be needed to analyze the binding kinetics, thus performing much longer simulations.

## 3. Conclusions

The N-terminal domain of SARS-CoV-2 spike protein is characterized by some insertion with respect to the SARS-CoV homologous one, and the surface regions generated by these residues have acquired the capability to bind sialic acid molecule (24, 26, 27). However, at present, the atomic details of the molecular complex is still missing.

Here, we address this issue through an extensive molecular dynamics simulation, where we were able to observe spontaneous binding events occurring between the spike protein of SARS-CoV-2 and the sialic acid molecule. In particular, we simulated the free motion of both the sialic acid molecule and the spike protein for times long enough to detect several binding between the two molecules. The main advantage of this approach is to consider exhaustively the physics of the system, taking into consideration electrostatic interactions as well as solvent and entropic effects.

We identify 5 sialic acid-interacting regions belonging to the NTD of the spike protein.

It has to be noted that physiologically the Spike protein is extensively glycosylated, a property that often escape the experimental characterization. Indeed, each Spike protomer has 22 glycosylation sites, which are supposed to affect which cells the virus can infect (32–35). This glycan shield, beyond being structurally necessary to bind ACE2, allows the virus to elude the host immune response (36). Importantly, none of the residues we found to be involved in sialic acid contact are in close proximity to these glycosylation sites, and therefore the binding region we identified could be considered free and capable to interact with sialic acid.

Moreover, since sialic acid is negatively charged, it is remarkable that the selected interacting regions are never characterized by negative net charge. The binding mode 2 is characterized by the highest positive charge that make it a suitable region to recognize the sialic acid molecule, and it is probably because of this that it is the most populated cluster among the five we proposed. Indeed, even looking at the sialic acid experimental binding region observed in MERS-CoV Spike protein, it emerges that the residues involved generate an overall charge of +1 (in unit of electron charge), similar to those characterizing our proposed binding modes.

Dynamic and structural analyses on these binding sites allow us to characterize the regions most prone to binding with the molecule. In particular, we consider the binding region 4 unsuitable for binding to sialic acid since binding occurs several times but of short duration. In addition, the residues corresponding to this region have a lower degree of correlation of their motion and the binding of the sialic acid increases the atomic mobility. On the other hand, three regions (number 1, 2, and 5) are particularly interesting because they have a high average correlation in terms of the motion of the residues that compose them and undergo an evident stabilizing effect after binding with sialic acid.

## 4. Materials and Methods

### 4.1. Datasets

- Unbound SARS-CoV-2 spike protein: modeled by I-TASSER server (37). The Top 10 structural analogs in PDB [as identified by TM-align (38)] are: 5x58, 6nzk, 6nb3, 3jcl, 5i08, 6cv0, 5szs, 5wrg, 6utk, 6b7n.
- *N*-Acetylneuraminic acid molecule: structure from PubChem database (id: 439197) (39). The parameters to perform the molecular dynamics simulation were obtained from the SwissParam server (40).

### 4.2. Molecular Dynamics Simulations

All simulations were performed using Gromacs 2019.3 (41). Topologies of the system were built using the CHARMM-27 force field (42). The protein was placed in a dodecahedric simulative box, with periodic boundary conditions, filled with TIP3P water molecules (43). For all simulated systems, we checked that each atom of the proteins was at least at a distance of 1.1nm from the box borders. Each system was then minimized with the steepest descent algorithm. Next, a relaxation of water molecules and thermalization of the system was run in NVT and NPT environments each for 0.1ns at 2fs time-step. The temperature was kept constant at 300K with v-rescale thermostat (44); the final pressure was fixed at 1bar with the Parrinello-Rahman barostat (45).

LINCS algorithm (46) was used to constraint bonds involving hydrogen atoms. A cut-off of 12 was imposed for the evaluation of short-range non-bonded interactions and the Particle Mesh Ewald method (47) for the long-range electrostatic interactions. The described procedure was used for all the performed simulations. In the following, we provide further details, specific of each simulation.

#### 4.2.1. SARS-CoV-2 Spike Trimer

Simulation of the SARS-CoV-2 spike trimeric complex was performed starting from the model structure proposed by the I-Tasser server (48). The addition of 3 sodium counterions rendered the systems electroneutral. The water density was of 1, 004kg/m^3^, close to the experimental value. The system was simulated with a 2fs time-step for 140ns in periodic boundary conditions.

#### 4.2.2. SARS-CoV Spike-2 N-Terminal Domain (Residue Range: 1–290)

We simulate also the N-terminal region of the SARS-CoV-2 spike in the presence of one molecule of sialic acid (neu5ac) in solution. We selected the domain ranging from residue 1 to 290 of the chain A of the trimeric complex. We added 2 Cl molecules to neutralize the system, while water density was 1, 008kg/m^3^. The simulation was carried out for 1.75μs with time steps of 2fs.

### 4.3. Principal Component and Clustering Analysis

We performed a PCA on the covariances of the C_α_ of the residues belonging to the peaks reported in Table 1 (111 atoms) and on the heavy atoms of the sialic acid molecule (21 atoms) using Gromacs 2019.3 internal packages. The covariances were calculated sampling the frames of the molecular dynamics trajectory each 500ps, and selecting only those frames where the sialic acid contacts at least one of the residues belonging to the peaks. A contact is defined if the center of mass of the sialic acid is at a distance lower than 6 from any heavy atom of the residue. We ended up with 1574 contact frames. In order to obtain the clusters showed in Figure 3A, we first evaluated the density of the points in the PC1-PC2 plane; then we filtered the points according to a density threshold and performed hierarchical clustering. By varying the threshold, we ended up with 5 groups on the density grid. We then reassigned the points of the original essential plane according to the position they had on the density grid (see Table 2). Analyses were performed using R standard libraries (49).

## Data Availability Statement

The original contributions presented in the study are included in the article/Supplementary Material, further inquiries can be directed to the corresponding author/s.

## Author Contributions

EM and GR conceived the research. LB performed molecular dynamics simulations. LB, MM, LD, and EM performed calculations and computational analysis. All authors analyzed the results, wrote, and revised the paper.

## Conflict of Interest

The authors declare that the research was conducted in the absence of any commercial or financial relationships that could be construed as a potential conflict of interest.
