# Supplementary material for: Exploring the Association Between Sialic Acid and SARS-CoV-2 Spike Protein Through a Molecular Dynamics-Based Approach
Source: Front Med Technol. 2021 Jan 13;2:614652. doi: 10.3389/fmedt.2020.614652 (PMC8757799; doi:10.3389/fmedt.2020.614652)
Supplement: Supplementary file 1 [file Data_Sheet_1.pdf]

Supporting Information: Exploring the association between sialic acid and SARS-CoV-2 spike protein through a molecular dynamics-based approach

Leonardo Bò,[1] Mattia Miotto,[2,1] Lorenzo Di Rienzo,[1] Edoardo Milanetti*,[2,1] and Giancarlo Ruocco[2,1]

[1]*Center for Life Nanoscience, Istituto Italiano di Tecnologia, Viale Regina Elena 291, 00161, Rome, Italy*
[2]*Department of Physics, Sapienza University, Piazzale Aldo Moro 5, 00185, Rome, Italy*

I. RESULTS

Comparison Between the Simulations of Spike N-terminal Domain Alone or in Trimeric Form.

The comparison between the RMSD of the domain simulated alone or inside the trimeric form of spike was done to verify that the interdomain interactions are not essential for the fold maintenance and the N-Terminal region of spike can be simulated alone, without undergoing major structural modifications. This allows us to lighten the computational cost of our simulation. However, this result does not demonstrate that the conformations obtained with the two different molecular dynamics are interchangeable.

To investigate this aspect we performed the RMSF analysis shown in Fig.1c of the Main Text, where we computed the mean fluctuations characterizing each residue during the molecular dynamics, both where the trimeric form of spike and the domain only were included in the simulations. This analysis shows which are the most mobile residues and the results obtained from the different simulations are very similar, proof of the similarity even of the internal motion of the spike N-terminal domains. Indeed, calculating the Pearson correlation coefficient between the RMSF values obtained in the residues of the three N-terminal domain of the whole spike and of the single domain simulation, we got the results shown in the Supporting Table I.

	NTD chain A	NTD chain B	NTD chain C	NTD alone
NTD chain A	1	0.695	0.766	0.619
NTD chain B	0.695	1	0.786	0.615
NTD chain C	0.766	0.786	1	0.646
NTD alone	0.619	0.615	0.646	1

Supporting Table I: Pearson correlation between the residues RMSF values calculated when the N-terminal domain of Spike protein is simulated inside the trimeric form or alone.

To further clarify this aspect, we studied also the correlation of the residues motions in all the domain we analyzed. For the 4 domains we build the correlation matrix, where in (i,j) cell are reported the correlation between the residue i and the residue j positions during the simulations. In Supporting Figure 1 we reported the colormap representing these matrices, showing an overall similarity.

To quantify this aspect, that is the similarity between the correlation of the residues motion in different simulations, we calculated the Pearson correlation between the values obtained in the simulation with the domain alone and the values obtained in the simulation with the trimer. We report the results in Supporting Table II

	NTD chain A	NTD chain B	NTD chain C
NTD alone	0.40	0.23	0.44

Supporting Table II: Pearson correlation between couples of residues motion correlation.

where all the p-values are significant (lower than 10^{-16}). In light of these considerations, we can conclude that not only the overall fold and the most mobile residues are conserved if the single domain are simulated, but also the main correlation between the residues motion are however present.

* Corresponding author: edoardo.milanetti@uniroma1.it

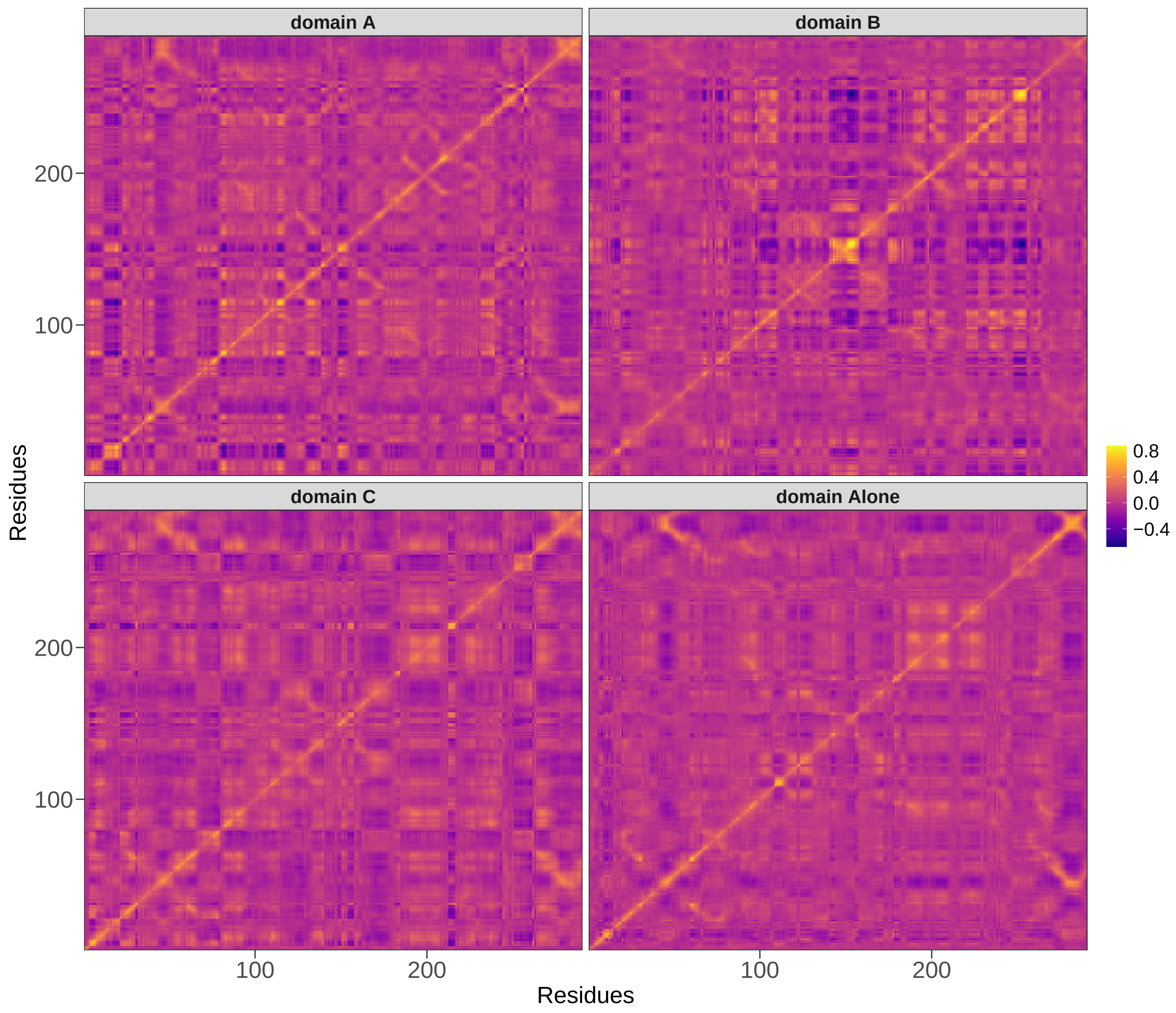

Supporting Figure 1: Colormap of correlations between residues. In (i,j) cell are reported the correlation between the residue i and the residue j positions during the simulations.

Binding-UnBinding Events

We evaluated the distances between the 5 main binding regions we identified using the PCA analysis on both the spike and sialic acid atoms coordinates. Computing the distances between the protein residues closer to the sialic acid molecule in each of the 5 binding modes, we obtained the Supporting Table III reporting the distances between the binding sites on Spike protein:

Binding Site	1	2	3	4	5
1	—	25.3 Å	43.7 Å	42.3 Å	40.1 Å
1	—	—	22.4 Å	20.2 Å	29.5 Å
1	—	—	—	5.2 Å	18.9 Å
1	—	—	—	—	22.2 Å
1	—	—	—	—	—

Supporting Table III: Distances between the 5 identified sialic acid binding sites on Spike protein N-terminal domain.

As shown in Supporting Table III, the binding sites are far, so as when an unbinding event occurs, the sialic acid has to explore a large part of spike protein to find another protein region to sit in. The only exception is the low distance registered between binding mode 3 and 4, that are indeed partially overlapping regions. In the following plot (Supporting figure 2) we highlighted the time spent by sialic acid molecule in each of the 5 binding mode during the simulation: it is interesting to note how it is probable the transition between the binding mode 3 and 4, characterized, as said, by a close proximity.

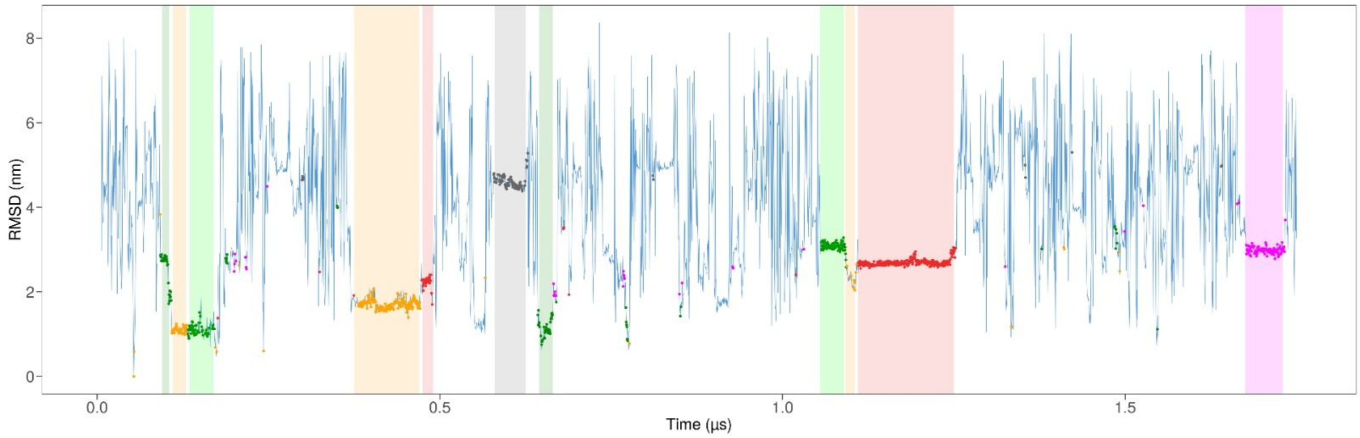

Supporting Figure 2: RMSD of the sialic acid molecule atomic positions as a function of simulation time. We used the first bound configuration as a reference. The frame the ligand spent in a bound configuration is highlighted.

With respect to Supporting Figure 2, in the following table (Supporting Table IV) we thus reported the time succession of binding modes occurred during the molecular dynamics, and each binding event is characterized with each life-time.

**Binding Mode**	**Color**	**Life-Time [ns]**
4	Green	10
3	Orange	20
4	Green	35
3	Orange	95
2	Red	15
1	Gray	45
4	Green	20
4	Green	35
3	Orange	13
2	Red	140
5	Magenta	55

Supporting Table IV: Succession of binding events, with the respective binding mode and life-time.

As it can be noted, the average life-time of a binding event is about 44 ns, ranging from 10 to 140. It is worth noting that the longest binding event (140 ns) occurs in the binding mode 2, the one characterized by a high positive net charge and that can be considered very suitable for sialic acid recognition.

II. METHODS

A. Molecular dynamics sialic acid parameters

The parameters used for the Spike protein are those implemented in CHARMM-27 force field [1]. The following parameters for the sialic acid molecule are obtained using the SwissParam web-server [2], where the input file was downloaded from PubChem database [3]

```
BONDS
OR   CR    363.214     1.4180
OR   HOR   560.905     0.9720
OR   C=O   417.476     1.3550
OR   HOCO  532.766     0.9810
O=C  C=O   931.963     1.2220
NC=O CR    335.651     1.4360
NC=O C=O   419.491     1.3690
NC=O HNCO  479.511     1.0150
CR   CR    306.432     1.5080
CR   C=O   301.539     1.4920
HCMM CR    342.991     1.0930

ANGLES
CR   OR   CR      86.143    106.9260
CR   OR   HOR     57.069    106.5030
C=O  OR   HOCO    41.956    111.9480
CR   NC=O C=O     59.084    119.6000
CR   NC=O HNCO    39.725    120.0660
C=O  NC=O HNCO    41.380    120.2770
NC=O CR   CR      75.564    109.9600
NC=O CR   HCMM    53.255    107.6460
CR   CR   CR      61.243    109.6080
CR   CR   HCMM    45.770    110.5490
OR   CR   CR      71.390    108.1330
OR   CR   HCMM    56.205    108.5770
HCMM CR   HCMM    37.134    108.8360
OR   CR   OR      83.192    111.3680
OR   CR   C=O     37.998    104.1120
CR   CR   C=O     55.917    107.5170
OR   C=O  O=C     83.120    124.4250
OR   C=O  CR      75.060    109.7160
O=C  C=O  CR      67.504    124.4100
O=C  C=O  NC=O    65.273    127.1520
NC=O C=O  CR      70.814    112.7350
C=O  CR   HCMM    46.778    108.3850

DIHEDRALS
OR   CR   CR   NC=O     0.150  3     0.00
OR   CR   CR   CR      -0.344  1     0.00
OR   CR   CR   CR       0.879  2   180.00
OR   CR   CR   CR       0.238  3     0.00
OR   CR   CR   HCMM    -0.327  1     0.00
OR   CR   CR   HCMM     0.536  2   180.00
OR   CR   CR   HCMM     0.140  3     0.00
OR   CR   CR   OR       0.204  1     0.00
OR   CR   CR   OR       0.699  2   180.00
OR   CR   CR   OR       0.480  3     0.00
OR   CR   OR   HOR      0.744  1     0.00
OR   CR   OR   HOR     -1.700  2   180.00
OR   CR   OR   HOR     -0.160  3     0.00
OR   CR   C=O  OR       0.224  1     0.00
OR   CR   C=O  OR       0.326  2   180.00
OR   CR   C=O  OR       0.159  3     0.00
OR   CR   C=O  O=C     -0.198  1     0.00
```

OR	CR	C=O	O=C	0.365	2	180.00
OR	CR	C=O	O=C	-0.070	3	0.00
OR	CR	OR	CR	0.115	1	0.00
OR	CR	OR	CR	-0.355	2	180.00
OR	CR	OR	CR	0.361	3	0.00
OR	C=O	CR	CR	-0.059	1	0.00
OR	C=O	CR	CR	-0.167	2	180.00
OR	C=O	CR	CR	0.101	3	0.00
O=C	C=O	OR	HOCO	0.831	1	0.00
O=C	C=O	OR	HOCO	3.076	2	180.00
O=C	C=O	OR	HOCO	-0.029	3	0.00
O=C	C=O	CR	CR	0.412	1	0.00
O=C	C=O	CR	CR	0.070	2	180.00
O=C	C=O	CR	CR	0.163	3	0.00
O=C	C=O	NC=O	CR	-0.160	1	0.00
O=C	C=O	NC=O	CR	3.147	2	180.00
O=C	C=O	NC=O	CR	-0.073	3	0.00
O=C	C=O	NC=O	HNCO	0.718	1	0.00
O=C	C=O	NC=O	HNCO	2.487	2	180.00
O=C	C=O	NC=O	HNCO	-0.227	3	0.00
O=C	C=O	CR	HCMM	0.330	1	0.00
O=C	C=O	CR	HCMM	-0.704	2	180.00
O=C	C=O	CR	HCMM	0.154	3	0.00
NC=O	CR	CR	CR	0.150	3	0.00
NC=O	CR	CR	HCMM	0.213	3	0.00
NC=O	C=O	CR	HCMM	-0.206	1	0.00
NC=O	C=O	CR	HCMM	0.346	2	180.00
NC=O	C=O	CR	HCMM	0.043	3	0.00
CR	NC=O	C=O	CR	0.324	1	0.00
CR	NC=O	C=O	CR	3.079	2	180.00
CR	NC=O	C=O	CR	0.254	3	0.00
CR	CR	OR	CR	-0.341	1	0.00
CR	CR	OR	CR	0.378	2	180.00
CR	CR	OR	CR	0.378	3	0.00
CR	CR	CR	CR	0.051	1	0.00
CR	CR	CR	CR	0.341	2	180.00
CR	CR	CR	CR	0.166	3	0.00
CR	CR	CR	HCMM	0.320	1	0.00
CR	CR	CR	HCMM	-0.315	2	180.00
CR	CR	CR	HCMM	0.132	3	0.00
CR	CR	OR	HOR	0.135	2	180.00
CR	CR	OR	HOR	0.118	3	0.00
CR	OR	CR	C=O	0.100	3	0.00
CR	CR	NC=O	C=O	-0.513	1	0.00
CR	CR	NC=O	C=O	0.347	2	180.00
CR	CR	NC=O	C=O	0.474	3	0.00
CR	CR	NC=O	HNCO	0.276	1	0.00
CR	CR	NC=O	HNCO	-0.190	2	180.00
CR	CR	NC=O	HNCO	0.163	3	0.00
CR	CR	CR	C=O	0.033	1	0.00
CR	CR	CR	C=O	-0.078	2	180.00
CR	CR	CR	C=O	0.071	3	0.00
CR	OR	CR	HCMM	0.285	1	0.00
CR	OR	CR	HCMM	0.160	2	180.00
CR	OR	CR	HCMM	0.285	3	0.00
CR	C=O	OR	HOCO	-0.583	1	0.00
CR	C=O	OR	HOCO	2.539	2	180.00
CR	C=O	OR	HOCO	-0.273	3	0.00
C=O	CR	OR	HOR	-0.826	1	0.00
C=O	CR	OR	HOR	-0.830	2	180.00
C=O	CR	OR	HOR	0.141	3	0.00
C=O	CR	CR	HCMM	-0.128	1	0.00
C=O	CR	CR	HCMM	0.029	2	180.00
C=O	NC=O	CR	HCMM	-1.050	1	0.00

```
C=O  NC=O CR   HCMM     0.681  2   180.00
C=O  NC=O CR   HCMM     0.011  3     0.00
CR   C=O  NC=O HNCO    -0.147  1     0.00
CR   C=O  NC=O HNCO     2.902  2   180.00
CR   C=O  NC=O HNCO     0.671  3     0.00
HNCO NC=O CR   HCMM    -0.308  1     0.00
HNCO NC=O CR   HCMM     0.137  3     0.00
HOR  OR   CR   HCMM     0.298  1     0.00
HOR  OR   CR   HCMM    -0.138  2   180.00
HOR  OR   CR   HCMM     0.173  3     0.00
HCMM CR   CR   HCMM     0.142  1     0.00
HCMM CR   CR   HCMM    -0.693  2   180.00
HCMM CR   CR   HCMM     0.157  3     0.00

IMPROPER
CR   CR   OR   C=O      0.000  0     0.00
CR   CR   OR   OR       0.000  0     0.00
C=O  OR   CR   O=C     10.147  0     0.00
CR   CR   OR   CR       0.000  0     0.00
CR   CR   OR   HCMM     0.000  0     0.00
CR   CR   CR   OR       0.000  0     0.00
CR   OR   CR   HCMM     0.000  0     0.00
CR   NC=O CR   CR       0.000  0     0.00
CR   NC=O CR   HCMM     0.000  0     0.00
NC=O C=O  CR   HNCO    -1.439  0     0.00
C=O  O=C  NC=O CR       9.284  0     0.00
CR   CR   CR   HCMM     0.000  0     0.00
CR   HCMM C=O  HCMM     0.000  0     0.00

NONBONDED nbxmod  5 atom cdiel shift vatom vdistance vswitch -
cutnb 14.0 ctofnb 12.0 ctonnb 10.0 eps 1.0 e14fac 1.0 wmin 1.5
OR      0.000000  -0.152100     1.770000
O=C     0.000000  -0.120000     1.700000   0.000000  -0.120000     1.400000
NC=O    0.000000  -0.200000     1.850000
CR      0.000000  -0.055000     2.175000   0.000000  -0.010000     1.900000
C=O     0.000000  -0.110000     2.000000
HNCO    0.000000  -0.046000     0.224500
HOR     0.000000  -0.046000     0.224500
HOCO    0.000000  -0.046000     0.224500
HCMM    0.000000  -0.022000     1.320000
```

[1] B. R. Brooks, C. L. Brooks, A. D. Mackerell, L. Nilsson, R. J. Petrella, B. Roux, Y. Won, G. Archontis, C. Bartels, S. Boresch, et al., Journal of Computational Chemistry **30**, 1545 (2009).

[2] V. Zoete, M. A. Cuendet, A. Grosdidier, and O. Michielin, Journal of Computational Chemistry **32**, 2359 (2011), URL `https://doi.org/10.1002/jcc.21816`.

[3] S. Kim, J. Chen, T. Cheng, A. Gindulyte, J. He, S. He, Q. Li, B. A. Shoemaker, P. A. Thiessen, B. Yu, et al., Nucleic Acids Research **47**, D1102 (2018), URL `https://doi.org/10.1093/nar/gky1033`.
